# Prognostic value of heparin-binding protein for mortality in severe COVID-19 pneumonia

**DOI:** 10.2217/bmm-2022-0265

**Published:** 2022-09-02

**Authors:** Tarık Acar, Birsen Ertekin, Mehmet Yortanlı, Sedat Koçak

**Affiliations:** ^1^Department of Emergency, University of Health Sciences, Beyhekim Training & Research Hospital, Konya, 42060, Turkey; ^2^Department of Emergency, Numune State Hospital, Konya, Turkey; ^3^Department of Emergency, Necmettin Erbakan University Meram Faculty of Medicine, Konya, Turkey

**Keywords:** biomarkers, COVID-19, diagnosis, emergency medicine, heparin-binding protein, infection, inflammation, mortality, pneumonia, prognosis

## Abstract

**Aim:** The study investigated heparin-binding protein (HBP) levels in patients with severe COVID-19 pneumonia and their relation to prognosis. **Methods:** A total of 134 patients with serious COVID-19 pneumonia were prospectively analyzed. HBP levels were statistically compared between both the patient and healthy control groups and within the patient group itself. **Results:** HBP was defined to be significantly higher in the patient group compared with the control group. There was a statistically significant distinction between the patients who survived and those who died with regard to HBP levels. When the cutoff value of HBP was >13.47, sensitivity (89.8%), specificity (74.1%) had area under the curve values of 0.806 (p < 0.001). **Conclusion:** HBP level may be used for prognosis prediction of patients with COVID-19.

COVID-19 usually manifests as an asymptomatic or moderate upper respiratory tract infection; however, approximately 5–10% of the patients may need intensive care due to severe pneumonia, acute respiratory distress syndrome (ARDS), sepsis, septic shock or multiple organ dysfunction syndrome (MODS) [1]. Uncontrolled pulmonary infiltration is the most evident clinical manifestation of the inflammatory response in the course of COVID-19. The resulting compensatory anti-inflammatory response is a mechanism that may be responsible for secondary infection and sepsis [2].

Sepsis is the clinical condition that leads to organ dysfunction resulting from the uncontrolled inflammatory response of the host against the infection [3]. Although sepsis usually develops due to bacterial infection, fungi or viruses may also lead to this condition. Most COVID-19 patients with sepsis do not have a bacterial superinfection, abut there is usually an irregular host response accompanied by evident hyperinflammation and immune suppression [2]. Hence, severe COVID-19 meets the definition of Sepsis-3 [4], revised for sepsis and septic shock, and the current markers used for negative outcomes in septic patients may also be used [5]. Experimental and clinical evidence support that heparin-binding protein (HBP) level is a valuable diagnostic marker in suspicion of sepsis and has an important role in the pathophysiology of organ dysfunction [6].

Neutrophils have been suggested to play an important role in the pathophysiology of COVID-19 [7]. HBP, also known as azurocidin or CAP37, is a protein stored in secretory vesicles and azurophilic granules in neutrophils. The easy mobilization of a large fraction of HBP from neutrophils and the other functions of HBP indicate the significance of this protein in early inflammation [8]. HBP, which is both a potent increaser of endothelial permeability and an inducer of inflammation, predicts pulmonary and renal dysfunction in septic patients [9]. As in bacterial infections, the HBP levels were also found to be high in viral infections such as influenza A (H1N1) and COVID-19 [5,10]. Therefore, the present study aimed to investigate the HBP levels in patients with severe COVID-19 pneumonia, its relationship with the prognosis and its correlation with other markers.

## Materials & methods

### Study design

This was a prospective cross-sectional cohort study conducted at the emergency department (ED) of a pandemic hospital between 1 November 2021 and 1 January 2022.

### Patient & control groups

Consecutive patients who had been admitted to the ED of the University of Health Sciences, Beyhekim Training and Research Hospital, between the aforementioned dates; diagnosed with severe COVID-19 pneumonia based on clinical, hematological, biochemical, microbiological and radiological examinations; who fulfilled the inclusion criteria; and who gave consent were evaluated prospectively. Age, gender, vital signs on admission, Glasgow Coma Scale score, the Systemic Inflammatory Response Syndrome and Quick Sequential Organ Failure Assessment scores were recorded on the form prepared by the researcher [11]. Arterial blood gases and blood samples for hematological and biochemical analyses were obtained from the patients in ED. Diagnosis of COVID-19 was verified with the real-time reverse transcriptase polymerase chain reaction test and lung involvement was verified with unenhanced CT scan of the thorax. Duration of hospital stay, need for vasopressor support and mechanical ventilation (noninvasive, invasive or high-flow nasal cannula oxygen) and outcomes (discharge or in-hospital mortality) were also recorded.

The diagnoses of severe COVID-19 pneumonia, sepsis, respiratory and circulatory insufficiency were verified in accordance with current guidelines [4,12,13]. Patients the whose CT thorax scan findings were reported to be consistent with COVID-19 pneumonia and whose polymerase chain reaction test results were positive were included in the study [14].

Patients who were under age 18, pregnant, immune-suppressive, had cancer, had chronic hepatic failure and renal failure, who had been subjected to trauma, who were found to have positive culture results and who did not sign the informed consent form were excluded from the study.

Forty-six volunteers in a similar age group who were completely healthy and had no chronic illness comprised the control group. The HBP levels were statistically compared between the patient and the control groups and between surviving and the deceased patient groups. The combination and correlation of HBP levels with other markers were also evaluated.

### Hematological & biochemical analyses

The blood samples taken from the ED were tested for white blood cell (WBC) count, neutrophil, lymphocyte, platelet, urea, aspartate aminotransferase (AST), alanine aminotransferase (ALT), troponin I, D-dimer, C-reactive protein (CRP), procalcitonin (PCT), albumin and lactate values. In patients whose COVID-19 diagnosis had been confirmed, an additional 10-cc venous blood sample was taken into EDTA tubes, centrifuged at 2000× *g* for 10 min, and the serum were separated. The serum samples were stored at -80 °C until the time of analysis. HBP levels were examined using the BT LAB ELISA kit and microELISA method (Nanhu Dist, Jiaxing, Zhejiang).

### Statistical analysis

Descriptive analyses were carried out. The categorical data were given as rates and numbers. The categorical data were compared with the chi-square test. The distribution of the numerical data was analyzed using the visual (histogram and likelihood plots) and analytical methods (Kolmogorov–Smirnov/Shapiro–Wilk tests). The normally distributed data were given as mean ± standard deviation and the nonnormally distributed data were given as median and interquartile range. The normally distributed groups were compared using the Student's t-test, and the nonnormally distributed groups were compared using the Mann–Whitney *U* test. The correlation coefficients of HBP with other markers and the statistical significance were calculated with the Spearman correlation test. For estimation of mortality, binary logistic regression analysis was carried out for determining the independent variables. The likelihood of mortality prediction of the HBP value and the regression models was analyzed with the receiver operating characteristic (ROC) curve analysis for prediction of mortality. In the assessment of the area under the curve (AUC), the diagnostic value of the test was interpreted to be statistically significant when the type 1 error level was below 5%. The AUC values of ROC analyses were compared with the DeLong method using the MedCalc program. Cutoff values were calculated with the Youden index. The sensitivity, specificity and negative predictive values (NPV) of the cutoff values were calculated. A p-level of <0.05 was accepted to be statistically significant in all tests. Statistical analyses were performed using the IBM SPSS version 22 and MedCalc version 20 programs.

## Results

**Figure 1.**
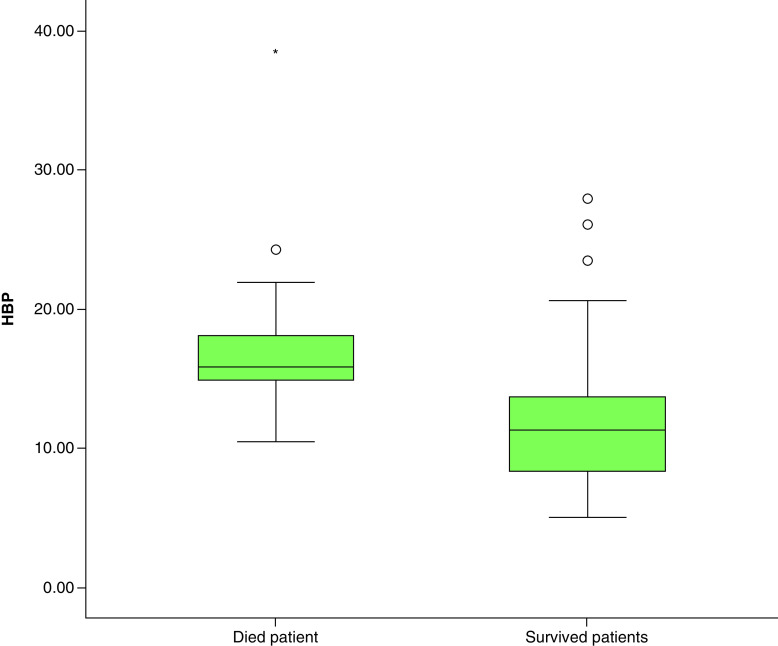
Comparison of heparin-binding protein for the surviving and deceased patient groups. HBP: Heparin-binding protein.

A total of 134 patients who had been admitted to the hospital between 1 November 2021 and 1 January 2022 were included in the study. Of the patients, 72 (%74.2) were males, and the mean age was 62.5 years (IQR 26). Of 134 patients, 85 survived and 49 died. The comparison of the surviving and the deceased patients with regard to demographic, clinical and laboratory data are presented in Table 1. When the dying and the surviving patients were compared with regard to age and gender, the mean age of dying patients was significantly higher (p < 0.001); however, no difference was found with regard to gender (p = 0.337). The temperature, pulse rate, respiratory rate, systemic inflammatory response syndrome, Quick Sequential Organ Failure Assessment, paCO_2_, urea, WBC, neutrophil, lymphocyte, AST, ALT, troponin I, D-dimer, CRP, PCT, lactate and HBP levels, need for MV and vasopressor drug were found to be significantly higher in the deceased patient group (p < 0.001 for all). Similarly, the oxygen saturation, systolic blood pressure, paO_2_, platelet, albumin levels and the Glasgow Coma Scale score were lower (p < 0.001 for all) and the duration of hospital stay was longer in the deceased patient group (p = 0.009). All patients of the COVID-19 cohort had bacterial cultures taken and none were positive. The patients were predominantly infected with the Delta variant of SARS-CoV-2 pathogen. Comparison of HBP for the surviving and deceased patient groups are presented in Figure 1.

**Table 1.** Comparison of demographic, clinical and laboratory findings of surviving and deceased patients.

Variables	All patients (n = 134)	Surviving patients (n = 85)	Deceased patient (n = 49)	p-value
Age, median (IQR), years	62.5 (26)	57 (27)	71 (25)	**<0.001**
Male, n (%)	72 (74.2)	43 (59.7)	29 (40.3)	0.337
Female, n (%)	62 (74.7)	42 (67.7)	20 (32.3)	
HBP, mean ± SD, ng/ml	13.73 ± 5.12	12.02 ± 4.86	16.7 ± 4.12	**<0.001**
Urea, median (IQR), mg/dl	86 (97)	64 (36)	162 (51)	**<0.001**
WBC, median (IQR),10^3^/ml	9.8 (7.5)	8.5 (3.7)	14 (6.7)	**<0.001**
Neutrophil, median (IQR), 10^3^/ml	11.6 (5.9)	9 (4.45)	15.4 (5.9)	**<0.001**
Lymphocyte, median (IQR), 10^3^/ml	1.2 (0.7)	1 (0.9)	1.3 (0.35)	**<0.001**
PLT, median (IQR), 10^3^/ml	190.5 (103)	201 (106)	155 (100)	**<0.001**
AST, median (IQR), U/l	49.7 (59)	37 (31)	90.8 (53)	**<0.001**
ALT, median (IQR), U/l	56 (59)	40 (37)	98 (59)	**<0.001**
Troponin I, median (IQR), ng/ml	42.5 (175)	31 (28)	163 (331)	**<0.001**
Albumin, median (IQR), g/l	31 (9.4)	34 (5.4)	25 (3.7)	**<0.001**
D-dimer, median (IQR), μg/ml	1887 (4084)	1334 (671)	6213 (6220)	**<0.001**
CRP, median (IQR), mg/l	54.8 (193.5)	5.3 (52.4)	224 (183.5)	**<0.001**
PCT, median (IQR), ng/ml	2.42 (14.75)	1.66 (1.09)	26.93 (32.16)	**<0.001**
Lactate, median (IQR), mmol/l	1.89 (1.2)	1.48 (0.6)	2.61 (0.5)	**<0.001**
PaCO2, median (IQR), mmHg	40 (10)	36 (7)	48 (12)	**<0.001**
PaO2, median (IQR), mmHg	62 (14)	65 (8)	53 (12)	**<0.001**
Saturation, median (IQR) %	75 (11)	76 (6)	65 (20)	**<0.001**
Respiratory rate, median (IQR), min	18.5 (10)	16 (4)	30 (9)	**<0.001**
Fever, median (IQR), °C	37 (1.3)	36.5 (1)	37.9 (0.7)	**<0.001**
Heart rate, median (IQR), min	85 (49)	74 (20)	125 (23)	**<0.001**
SBP, median (IQR), mmHg	124.5 (60)	140 (25)	77 (14)	**<0.001**
GCS, median (IQR)	13 (2)	14 (2)	11 (2)	**<0.001**
SIRS, median (IQR)	2 (1)	2 (1)	4 (1)	**<0.001**
qSOFA, median (IQR)	1 (2)	1 (0)	3 (1)	**<0.001**
MV support, n (%)	62 (46.3)	16 (25.8)	46 (74.2)	**<0.001**
Vasopressor support, n (%)	50 (37.3)	6 (12)	44 (88)	**<0.001**
Length of stay in hospital, median (IQR), day	15 (10)	14 (10)	17 (10)	0.009

Bold text: a p-level <0.05 was accepted to be statistically significant in all tests.

ALT: Alanine transaminase; AST: Aspartate transaminase; GCS: Glascow Coma Scale; HBP: Heparin-binding protein; IQR: Interquartile range; MV: Mechanical ventilation; PCT: Procalcitonin; PLT: Platelet; qSOFA: Quick Sequential Organ Failure Assessment; SBP: Systolic blood pressure; SIRS: Systemic Inflammatory Response Syndrome; WBC: White blood cell.

**Figure 2.**
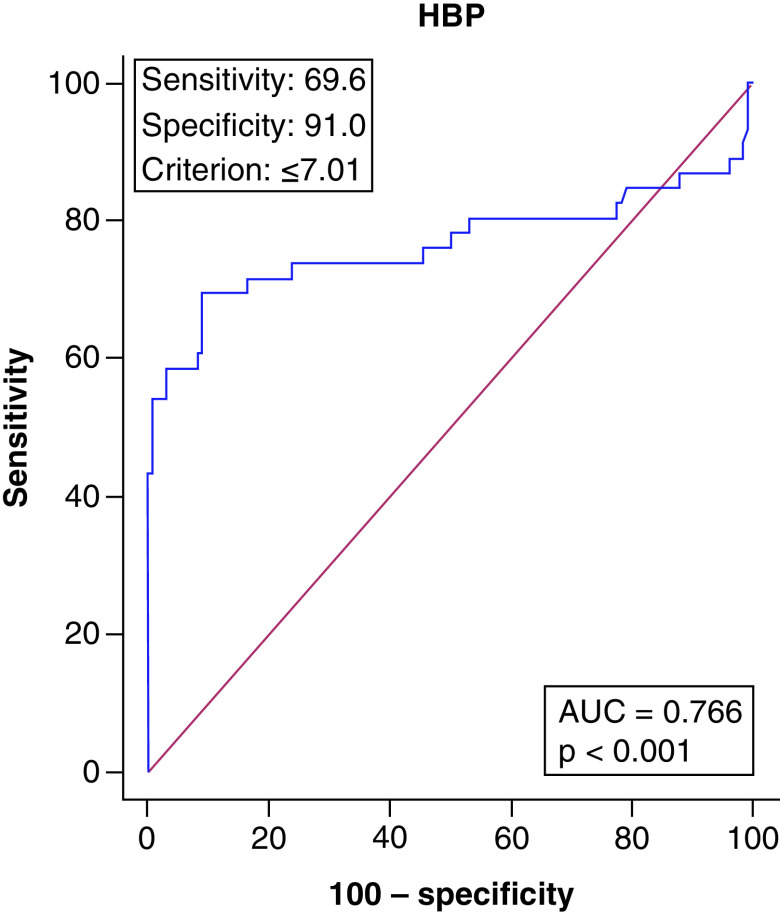
Receiver operating characteristic curve for patient and control groups in the heparin-binding protein values. AUC: Area under the curve; HBC: Heparin-binding protein.

**Figure 3.**
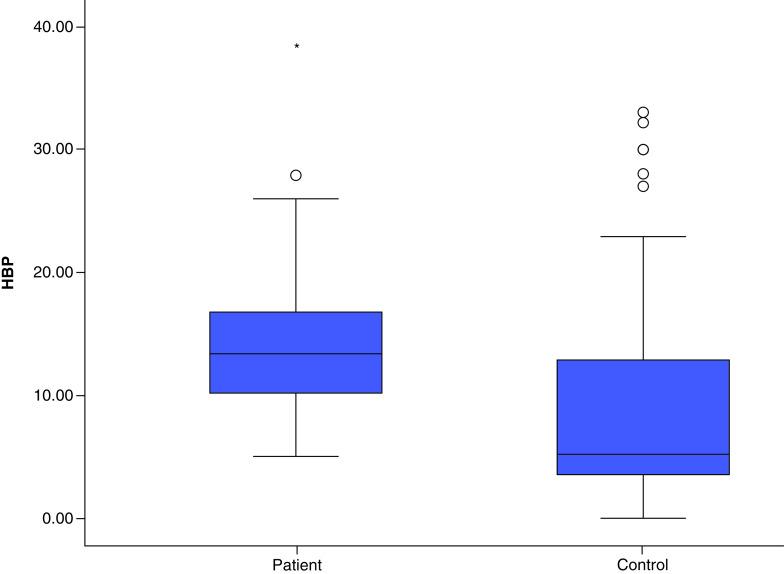
Comparison of heparin-binding protein values for the patient and control groups. HBC: Heparin-binding protein.

The comparison of the patient and the control groups with regard to age, gender and HBP levels is presented in Table 2. HBP was found to be significantly higher in the patient group (p = 0.002). In the ROC analysis, sensitivity was 69.6% and specificity was 91% when the HBP cutoff value was ≤7.01 (AUC: 0.766, p < 0.001) (Figure 2). Comparison of HBP for the patient and control groups is presented in Figure 3.

**Table 2.** Comparison of age, gender and heparin-binding protein values for patient and control groups.

Variables	Patient (n = 134)	Control (n = 46)	p-value
Age, median (IQR), years	62,5 (26)	46 (14)	**<0.001**
Male, n (%)	72 (74.2)	25 (25.8)	0.942
Female, n (%)	62 (74.7)	21 (25.3)	
HBP, mean ± SD, ng/ml	13.73 ± 5.12	9.12 ± 8.93	**0.002**

HBP: Heparin-binding protein; IQR: Interquartile range.

When the HBP levels were compared between patients receiving and not receiving MV support, they were found to be significantly higher among those requiring MV support (p = 0.003). The mean HBP level was 15.13 ± 5.19 ng/ml in patients receiving MV support and 12.53 ± 4.77 ng/ml in those not receiving MV support.

**Figure 4.**
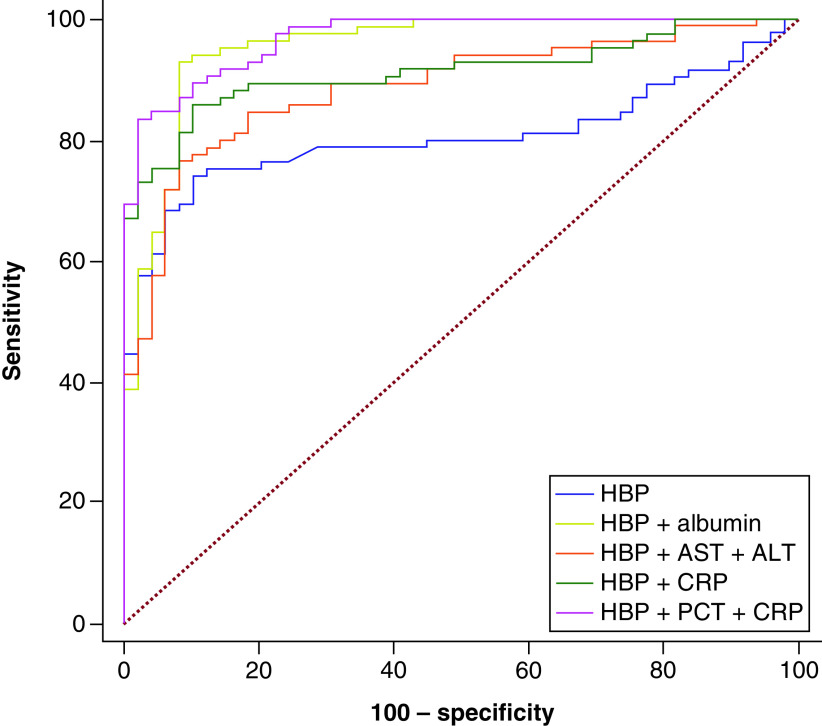
Receiver operating characteristic curve of heparin-binding protein and its combinations in predicting mortality. ALT: Alaninetransaminase; AST: Aspartate transaminase; CRP: C-reactive protein; HBC: Heparin-binding protein; PCT: Procalcitonin.

The ROC analysis of the prognostic value of HBP alone and HBP + AST + ALT, HBP + CRP, HBP + albumin, HBP + PCT + CRP combinations for mortality have been displayed in Figure 4. The AUC values of these parameters for mortality prognostication have been demonstrated in Table 3. Accordingly, the HBP + PCT + CRP combination had a higher AUC value compared to HBP alone, HBP + AST + ALT, HBP + CRP and HBP + albumin (0.970, 0.806, 0,888, 0.914, 0.955) (p < 0.001 for all).

**Table 3.** The receiver operating characteristic analysis of parameters in the prognostication of mortality.

Parameters	AUROC	95% CI		Cutoff	Sensitivity (%)	Specificity (%)	NPV	p-value
		Lower limit	Upper limit					
HBP	0.806	0.732	0.881	>13.47	89.8	74.1	92.6	<0.001
HBP + AST + ALT	0.888	0.905	0.983	>0.2643	91,8	75.3	94.1	<0.001
HBP + CRP	0.914	0.866	0.962	>0.3326	89.8	84.7	93.5	<0.001
HBP + albumin	0.955	0.919	0.991	>0.277	91.8	91.8	95.1	<0.001
HBP + PCT + CRP	0.970	0.948	0.993	>0.1549	98	82.4	98.6	<0.001

ALT: Alanine transaminase; AST: Aspartate transaminase; AUROC: Area under the receiver operating characteristic curve; CRP: C-reactive protein; HBP: Heparin-binding protein; NPV: Negative predictive value; PCT: Procalcitonin.

The sensitivity, specificity and NPV values for prognostication of mortality according to the cutoff values of HBP alone and HBP + AST + ALT, HBP + CRP and HBP + albumin, HBP + PCT + CRP combinations are presented in Table 3. Among all parameters, the sensitivity (98%), specificity (82.4%) and the NPV (98.6%) values reached a maximum when the cutoff value of HBP + PCT + CRP was >0.1549. When the cutoff value of HBP alone was >13.47, sensitivity (89.8%), specificity (74.1%) and NPV (92.6%) had the lowest values.

In the correlation analysis, a mild correlation was observed between HBP and albumin and CRP (r = -0.328 and 0.278, respectively; p < 0.001 for all). A moderate correlation was found between HBP and urea, neutrophil, lymphocyte count, AST, ALT (r = 0.528, 0.551, 0.461, 0.578 and 0.510, respectively; p < 0.001 for all). A strong correlation was determined between HBP and D-dimer, PCT and lactate (r = 0.784, 0.673 and 0.865, respectively; p < 0.001 for all) (Table 4).

**Table 4.** Correlation between heparin-binding protein and other markers in patients with severe COVID-19 pneumonia.

Markers	HBP Correlation coefficient	p-value
Urea	0.528	<0.001
Neutrophil	0.551	<0.001
Lymphocyte	0.461	<0.001
AST	0.578	<0.001
ALT	0.510	<0.001
Albumin	-0.328	<0.001
D-dimer	0.784	<0.001
CRP	0.278	<0.001
PCT	0.673	<0.001
Lactate	0.865	<0.001

ALT: Alanine transaminase; AST: Aspartate transaminase; CRP: C-reactive protein; HBP: Heparin-binding protein; PCT: Procalcitonin.

**Figure 5.**
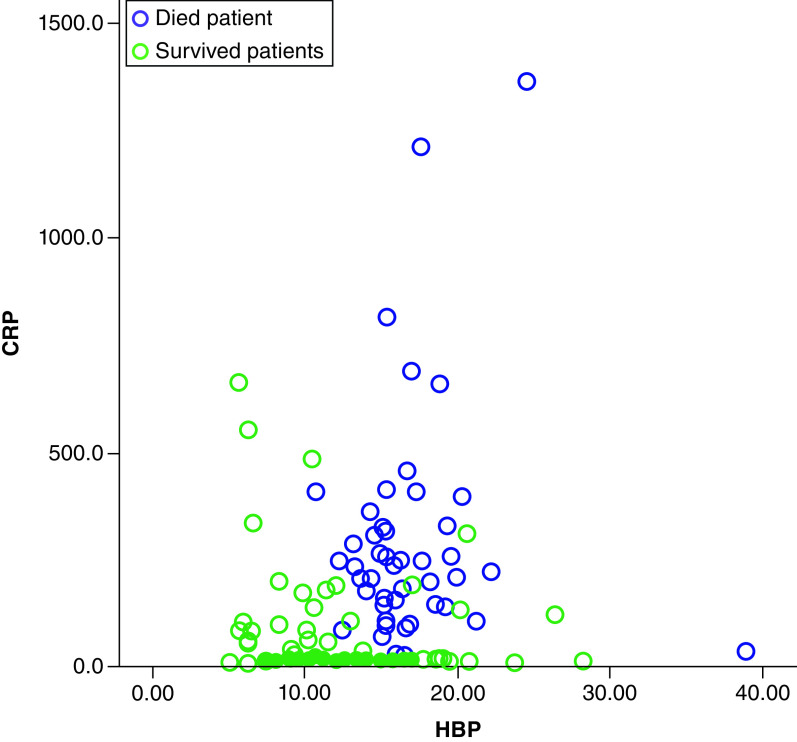
The scatter plot of heparin-binding protein and C-reactive protein for the surviving and deceased patient groups. CRP: C-reactive protein; HBC: Heparin-binding protein.

The regression analysis revealed that HBP + CRP, HBP + albumin and HBP + PCT + CRP combinations were independent predictors for mortality in patients with severe COVID-19 pneumonia (Table 5). The scatter plot of HBP and CRP for the surviving and deceased groups are displayed in Figure 5.

**Table 5.** Binary logistics regression analysis on the markers associated with mortality in patients with severe COVID-19 pneumonia.

Model	β	Standard error	RR (95% GA)	p-value
HBP	0.257	0.064	1.29 (1.41–1.47)	<0.001
CRP	0.011	0.002	1.01 (1.01–1.02)	<0.001

Model	β	Standard error	RR (95% GA)	p-value
HBP	0.239	0.070	1.27 (1.01–1.46)	0.001
Albumin	-0.555	0.105	0.57 (0.47–0.71)	<0.001

Model	β	Standard error	RR (95% GA)	p-value
HBP	0.212	0.073	1.24 (1.07–1.43)	0.004
PCT	0.232	0.064	1.01 (1.00–1.01)	0.001
CRP	0.008	0.002	1.01 (1.01–1.02)	<0.001

CRP: C-reactive protein; HBP: Heparin-binding protein; PCT: Procalcitonin; RR: Relative risk.

## Discussion

In severe COVID-19 cases, the immune pathogenesis and microcirculation disorders caused by cytokine storm are significant risk factors for viral sepsis, MODS, ARDS and high mortality [15]. Severe COVID-19 patients meet the diagnostic criteria for sepsis and septic shock [4], and COVID-19 was shown to be the only factor in most cases [16]. In a study investigating COVID-19 patients, 76% of whom had been diagnosed with sepsis, bacteria and fungus growth was not detected [2]. Hence, viral sepsis would be a correct definition for the clinical symptoms of severe COVID-19 patients [17]. Many studies have confirmed that plasma HBP levels increase in sepsis [18,19]. Paulsson *et al.* [20] reported that the HBP levels were significantly higher in patients with pneumonia. Saridaki *et al.* [5] also reported that the HBP levels of septic patients with COVID-19 pneumonia were higher than those without sepsis. The HBP levels in the severe COVID-19 pneumonia group were also significantly higher than those of healthy controls in the present study (p = 0.002). Furthermore, according to the ROC analysis results, sensitivity of 69.6%, specificity of 91% and an AUC value of 0.766 when the cutoff value was ≤7.01 confirm that HBP is a potential marker to be added to the current diagnostic tools in these patients.

Elevated HBP level was reported to be a good marker for mortality rates in MODS and critically ill patients [21]. In another study, HBP was found to be elevated hours before organ dysfunction development and HBP levels >30 ng/ml had 78% sensitivity for prognostication of severe sepsis [22]. In the study of Saridaki *et al.* [5] investigating patients with COVID-19 pneumonia, the authors reported that HBP could be used alone for prognostication of mortality with 79.5% and 84.6% sensitivities at 18 and 35 ng/ml cutoff values. In the present study, HBP levels were also found to be significantly higher in the deceased patient group compared with survivors (p < 0.001). In addition, HBP alone was shown to be a useful marker with 89.8% sensitivity, 74.1% specificity, 92.6% NPV and 0.806 AUC values when the cutoff value was >13.47.

Neutrophils play an important role in acute lung injury or ARDS development [23]. Patients with severe COVID-19 tend to have elevated neutrophil levels in blood and the lungs, and this condition suggests that they may be effective in the pathophysiology of COVID-19 [7]. HBP is stored in azurophilic granules and excretory vesicles of neutrophils and released rapidly and abundantly when neutrophils are activated [6]. The correlation of HBP with severe COVID-19 is an indicator of its role in systemic inflammation [24]. To show the value of HBP in the diagnosis of sepsis, it was compared with inflammation markers, PCT, IL-6, CRP, WBC and lactate [25]. In several studies conducted in recent years, it was recommended to use HBP together with other parameters such as PCT [26,27]. Saridaki *et al.* [5] associated the positive correlation between HBP and the neutrophil count with respiratory failure in COVID-19 patients. In the present study, HBP had a mild correlation with CRP, a moderate correlation with neutrophil and lymphocytes and a strong correlation with PCT (p < 0.001). This condition shows that HBP may have an important role in the pathophysiology of COVID-19, in addition to other inflammatory markers. Furthermore, this study indicates that the combination of HBP with other markers such as CRP and PCT increased its prognostic value compared with using it alone (AUC: 0.970 for HBP + PCT + CRP combination).

Respiratory failure and hypoxemia are the most severe complications of COVID-19 pneumonia and the main factors contributing to a poor prognosis [28]. Hypoxia was shown to trigger neutrophil activation and release of granular proteins of neutrophils [29]. Kaukonen *et al.* [10] stated that HBP values significantly differed between critically ill influenza A (H1N1) patients receiving and not receiving MV support. In another study, HBP levels were shown to be significantly higher in critically ill ARDS patients compared with those without ARDS [30]. Xue *et al.* [24] associated HBP levels with hypoxia-related pulmonary ventilation insufficiency in their study investigating severe COVID-19 patients. Saridaki *et al.* [5] also associated the correlation between HBP levels and the pO_2_/FiO_2_ ratio and lactate with hypoxia, shock and vascular endothelial dysfunction. In the present study, HBP levels were found to be higher in patients who required MV (p = 0.003). This result shows that elevated HBP may be associated with the severity of respiratory failure developing in severe COVID-19 pneumonia.

COVID-19 may trigger inflammatory storms that affect perfusion, and this may lead to MODS [31]. Several recent studies reported that levels of HBP can predict the organ dysfunction in patients with sepsis or stay in the intensive care unit [19,22,30]. Kahn *et al.* showed that HBP levels increase as the number of organ failures increases [19]. Mellhammar *et al.* reported that HBP levels increased significantly before the onset of organ dysfunction and decreased before discharge compared to admission levels in patients with severe COVID-19 (respectively, 22.4 ng/ml, 9.0 ng/m and 7.6 ng/ml). Also, according to the authors, due to its role in endothelial dysfunction, HBP may be used as a prognostic marker for organ dysfunction development in COVID-19 [32]. HBP was recently emphasized to be an important diagnostic marker in acute renal damage [33]. Saridaki *et al.* [5] associated HBP levels with organ dysfunction due to the presence of a positive correlation between HBP and serum creatinine levels in COVID-19 patients. In other studies, liver functions and albumin levels have been defined as important determinants of mortality in patients with severe COVID-19 [34,35]. In addition, considering the strong association between thromboembolism and COVID-19, myocardial damage, D-dimer and cardiac markers are also significant in the follow-up of these patients [36]. Xue *et al.* [24] reported a strong correlation between D-dimer, AST, ALT, creatine kinase, cardiac troponin I and myoglobin levels and HBP in patients with severe COVID-19. Many studies have indicated that the combination of HBP with other inflammatory markers is more valuable in predicting the long-term outcome of patients with sepsis [5,27,37]. Thus, levels of HBP was combined with albumin, AST and ALT, which were important indicators of mortality in COVİD-19 in the present study. Consistent with the literature, our study shows that HBP + albumin and HBP + AST + ALT combinations were more valuable than HBP alone in predicting mortality (respectively, AUC: 0.955, 0.888 and 0.806). Katsaros *et al.* stated that prognostic and diagnostic power of HBP in patients with sepsis may be due to a positive relationship with organ function markers (creatinine, bilirubin and lactate) and other inflammatory biomarkers (neutrophils, monocytes, PCT and CRP) [27]. On the other hand, Zanfaly *et al.* have stated that HBP is the best indicator of circulatory dysfunction in septic patients group which have statistically significant positive correlation between of HBP with lactate [38]. In the present study, we found that HBP had a mild negative correlation with albumin; a moderate correlation with urea, AST and ALT; and a strong correlation with lactate. Therefore, the HBP levels seem to be a useful marker for detecting organ dysfunction in these patients. However, our study which we have done with limited number of markers should support by studies analyzing more indicators of organ dysfunction.

## Limitations

This was a single-center study conducted with a small number of patients. The small sample may affect the interpretation of HBP levels. Therefore, the results should be confirmed through multicenter studies conducted with large samples. Second, previously used medications may interact with HBP, and this condition has an important prognostic value; however, we could not monitor the post-treatment HBP changes [18,39]. Furthermore, we did not use a statistical method to correct the effects of accompanying drugs. Third, we could only evaluate the in-hospital mortality. Hence, we do not have information regarding the prognostic value of HBP for long-term mortality rates. Fourth, in our study, we were able to compare the HBP levels of the patient group only with those of the healthy control group. However, comparison with other causes of viral and bacterial pneumonia of patient groups would be more appropriate for talking about diagnostic value of HBP in serious COVİD-19 pneumonia [19,26,32]. These limitations notwithstanding, we hope that the results of our study, which is one of the few studies investigating the value of HBP in severe COVID-19 prognosis, will contribute to the literature.

## Conclusion

HBP levels may be used as an additional marker for the diagnosis of patients with severe COVID-19 pneumonia. The results of our study support the potential of HBP for prognostication of mortality, whether used alone or in combination with other markers. In addition to the correlation with inflammatory markers, HBP was also found to be associated with organ dysfunction developing in severe COVID-19 pneumonia.

**Summary points**

- Heparin-binding protein (HBP) levels in the severe patient group were significantly higher than those of healthy controls in our study (p = 0.002). In the receiver operating characteristic analysis, the area under the curve was 0.766 when the HBP cutoff value was ≤7.01 (p < 0.001).
- When the HBP levels were compared between patients receiving and not receiving mechanical ventilation support, they were found to be significantly higher among those requiring mechanical ventilation support (p = 0.003).
- HBP levels were found to be significantly higher in the deceased patient group compared with survivors (p < 0.001).
- HBP alone was shown to be a useful marker with 0.806 area under the curve values for prognostication of mortality when the cutoff value was >13.47.
- Among all parameters, the sensitivity, specificity and negative predictive values reached a maximum when the cutoff value of HBP + procalcitonin + C-reactive protein was >0.1549.
